# Timeliness of infectious disease reporting, the Netherlands, 2003 to 2017: law change reduced reporting delay, disease identification delay is next

**DOI:** 10.2807/1560-7917.ES.2019.24.49.1900237

**Published:** 2019-12-05

**Authors:** Corien M Swaan, Albert Wong, Axel Bonačić Marinović, Mirjam EE Kretzschmar, Jim E van Steenbergen

**Affiliations:** 1 Centre for Infectious Disease Control, National Institute for Public Health and the Environment (RIVM), Bilthoven, the Netherlands; 2 Department of Statistics, Mathematical Modelling and Data Logistics, National Institute for Public Health and the Environment (RIVM), Bilthoven, the Netherlands; 3 Julius Center for Health Sciences and Primary Care, University Medical Center Utrecht, Utrecht University, Utrecht, the Netherlands; 4 Centre for Infectious Diseases, Leiden University Medical Centre, Leiden, the Netherlands

**Keywords:** disease notification, infectious disease reporting, surveillance system, timeliness, outbreak control

## Abstract

**Background:**

Timely notification of infectious diseases is essential for effective disease control and needs regular evaluation.

**Aim:**

Our objective was to evaluate the effects that statutory adjustments in the Netherlands in 2008 and raising awareness during outbreaks had on notification timeliness.

**Methods:**

In a retrospective analyses of routine surveillance data obtained between July 2003 and November 2017, delays between disease onset and laboratory confirmation (disease identification delay), between laboratory confirmation and notification to Municipal Health Services (notification delay) and between notification and reporting to the National Institute for Public Health and the Environment (reporting delay) were analysed for 28 notifiable diseases. Delays before (period 1) and after the law change (periods 2 and 3) were compared with legal timeframes. We studied the effect of outbreak awareness in 10 outbreaks and the effect of specific guidance messages on disease identification delay for two diseases.

**Results:**

We included 144,066 notifications. Average notification delay decreased from 1.4 to 0.4 days across the three periods (six diseases; p < 0.05), reporting delay decreased mainly in period 2 (from 0.5 to 0.1 days, six diseases; p < 0.05). In 2016–2017, legal timeframes were met overall. Awareness resulted in decreased disease identification delay for three diseases: measles and rubella (outbreaks) and psittacosis (specific guidance messages).

**Conclusions:**

Legal adjustments decreased notification and reporting delays, increased awareness reduced identification delays. As disease identification delay dominates the notification chain, insight in patient, doctor and laboratory delay is necessary to further improve timeliness and monitor the impact of control measures during outbreaks.

## Introduction

Effective communicable disease surveillance systems are a prerequisite to ensure early detection of health threats and their timely control. Delay in infectious disease reporting might hamper timely outbreak control measures, such as prophylaxis for contacts, active case finding or identifying and eliminating a common source. In the Netherlands, earlier studies revealed that up to 42% of infectious diseases reported between June 2003 and December 2008 were not notified within 3 days after laboratory confirmation, and there were substantial reporting delays for four of six investigated diseases [1,2]. 

**Figure 1:**
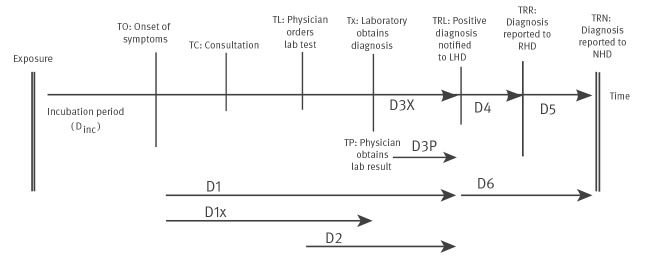
Notification and reporting chain for infectious diseases

D1: total local delay; D1X: disease identification delay; D2: total testing delay; D3X: laboratory notification delay; D3P: physician notification delay; D4: local reporting delay; D5: regional reporting delay; D6: reporting delay; LHD: local health department (NHS in the Netherlands); RHD: regional health department. NHD: national health department (RIVM in the Netherlands).

Infectious disease reporting is a process with several steps, the notification and reporting chain (Figure 1) [2]. Reporting delay on local level is the result of (i) the incubation time, (ii) the time until the patient decides to seek medical care, (ii) doctors’ delay in recognising the disease and initiating laboratory testing, (iv) delayed laboratory confirmation of the diagnosis and (v) delayed notifications by physicians and laboratories to the local health department (LHD) or Municipal Health Services (MHS) in the Netherlands, defined as notification delay. Subsequently, reporting delays from the LHD to regional and national health services (NHS), defined as reporting delay, influence timely detection of multiregional or national outbreaks.

Public health authorities stimulate early notification and reporting through provision of information and guidance to medical professionals. In addition, many, also European, countries have included timeframes for notification and reporting in their laws on notifiable diseases [3,4]. These legal requirements, which may even include penalties for non-adherence, are a strong instrument and an important step in the chain through which governments can control early detection and timely public health response. Nevertheless, legal requirements need careful consideration and evaluation, and other facilitating elements such as clear and uniform reporting timeframes, procedures and feedback on notifications are important as well [5].

In the Netherlands, legal adjustments were made to mandatory infectious disease reporting in December 2008 to reduce notification and reporting delays. Under the former Infectious Disease Act from 1998, diseases were notifiable by either physicians (group B notifiable diseases, D3P in Figure 1) or laboratories (group C diseases, D3X). When the new Public Health Act came into force in December 2008, both group B and C diseases became notifiable to the MHS for both physicians and laboratories [6]. The notification timeframe of 1 working day remained unchanged, likewise the timeframe for group A diseases, which require immediate notification upon disease suspicion either by physicians or laboratories. The timeframe for reporting from the MHS to the NHS, the National Institute for Public Health and the Environment (RIVM); D6 in Figure 1), was reduced for some group B and C diseases: from 7 to 3 days for hepatitis A, Q fever and psittacosis, and from 1 month to 7 days for pertussis and malaria. In this study, we evaluate whether the legal adjustments resulted in faster reporting and whether legal and outbreak control timeframes were met.

In order to address earlier steps in the notification and reporting chain such as delays in notification by doctors and laboratories, the RIVM raises outbreak awareness among MHS, physicians and microbiologists through a weekly signalling report sent by email. Further guidance, e.g. about the availability of laboratory tests and notification criteria, is also provided through an instant alert system, so-called inf@ct and labinf@ct email messages. Our second objective was to evaluate whether these awareness systems reduced reporting delays during outbreaks. 

## Methods

### Data selection

Since 2003, the MHS have been reporting all notified infectious diseases to the RIVM through a web-based application [7]. We performed a retrospective analysis of routine surveillance data and extracted data on all cases notified between July 2003 and November 2017. From those, we excluded notifications of Creutzfeldt–Jacob disease and tuberculosis, as the notification procedures were not comparable with the rest. Group A diseases (polio, smallpox, Middle East respiratory syndrome (MERS), severe acute respiratory syndrome (SARS) and viral haemorrhagic fever) were excluded as they were notifiable upon suspicion, before laboratory confirmation. Hepatitis C and chronic hepatitis B were excluded as date of disease onset in most cases was not known. We also excluded rare diseases with less than 10 notifications in the full study period. As a result, we included 19 notifiable diseases for the time period of validity of the former Act, until the end of 2008, and 28 diseases for the time period of validity of the new Act from 2009 onwards. For each case, date of symptom onset (T_O_), date of laboratory confirmation (T_X_), date of notification at the MHS (T_RL_) and date of reporting to the RIVM (T_RN_) were extracted. As the MHS did not provide all dates for every case, only cases that had dates available to calculate delays were included in the study.

### Calculation of delays

The following delays were calculated for each case, as visualised in Figure 1:

- **D1: total local delay**, delay between onset of disease and notification to the MHS (T_RL_ − T_O_)
- **D1X: disease identification delay**, delay between onset of disease and laboratory confirmation (T_X_ − T_O_)
- **D2: total testing delay,** delay between ordering laboratory test by physician and notification to the MHS (T_L_ – T_RL_)
- **D3: notification delay**, delay between laboratory confirmation and notification to the MHS (T_RL_ – T_X_)
- **D4: local reporting delay**, delay between notification to the MHS and reporting to the RHD (T_RR_ – T_RL_)
- **D5: regional reporting delay_,_** delay between reporting to the RHD and reporting to the NHD (T_RN_ – T_RR_)
- **D6: reporting delay**, delay between notification at the MHS and reporting to the NHD (T_RN_ – T_RL_)

Delays shorter than 0 or longer than 365 days were excluded, as a mistake in data entry was considered likely. In D3, weekend days were removed, as notifications are legally not obligatory in the weekend. Medians of D1X, D3 and D6 were calculated for all cases per disease and for all diseases together, for each of three periods: period 1 (P1): notification to MHS between July 2003 and January 2009 (former law), period 2 (P2) between January 2009 and January 2013 and period 3 (P3) between January 2013 and November 2017. We divided the period with the new law in two equal periods to analyse delay trends in time.

### Delay analysis

Median delays per disease per period, median delay of all cases per period and average across the median delays for the different diseases in period 2 and period 3 were compared with period 1, using the permutation test [8]. This test uses the data to construct a null distribution and derive p values without making any a priori assumption on the distribution of the data. This is particularly useful when we are dealing with strongly right-skewed distributions, as is the case with delay times. Statistical calculations were performed on medians and means. Since there was substantial overlap in outcomes, we chose to present the outcomes on medians for reasons of clarity and representativeness. As the number of notifications per disease varied widely, averages of medians of delays per diseases were calculated per period as well. Medians and boxplots were calculated per year for notification and reporting delay to study trends over time within periods. Percentage of six diseases notified after 3 days (including weekend days) of most recent notifications in 2016–2017 were calculated for comparison with percentages calculated by Reijn et al. for these diseases over period 1 [1].

### Timeliness analysis

Heads of laboratories and physicians need to notify a notifiable disease within 1 working day to the MHS. The MHS needs to report a notified disease within 1, 3 or 7 days, depending on the disease, to the RIVM. In order to present the most up-to-date situation, we used notifications of 2016–2017 and calculated the percentage of notified cases within the legal timeframes per disease.

Other timeframes were based on serial intervals and incubation periods, as the duration of these intervals determines how fast an outbreak develops. Midpoints of the ranges of incubation periods for 10 person-to-person transmissible diseases were retrieved from the national guidelines of the RIVM and medians of serial interval distributions for eight of these 10 diseases were retrieved from literature [2,9-11]. Lastly, we included a timeframe for outbreak control calculated by Bonačić et al. for six person-to-person transmissible diseases based on the proportion (PIR2) of expected new infections produced by each secondary case at the time of notification of the index case to the MHS [2]. An outbreak is controlled, in other words the incidence begins to decline, if the average number of cases produced by an infected person is < 1. The number of cases produced by each secondary case is PIR2 multiplied by the reproduction number. Therefore, outbreak control can be achieved if PIR2 × R < 1. The following total local delays (D1) were determined to achieve the outbreak control timeframe: 17 days for hepatitis A, 42 days for hepatitis B, 5 days for measles, 8 days for mumps, 4.5 days for pertussis and 3 days for shigellosis.

As performance threshold indicator, reporting was considered timely when at least 80% of cases were notified within the specific timeframe in a specific period, in line with the World Health Organization (WHO) Joint External Evaluation Tool which recommends at least 80% of all reporting units report in time [12]. In addition, RIVM uses 80% as the threshold for minimal timely reporting of D6 in feedback to the MHS.

### Increased awareness and guidance

To determine the effect of increased awareness and guidance for local health professionals on disease identification and notification delays, we identified the following outbreaks in our study period (2003–2017) which were addressed in the signalling reports and (laboratory)inf@cts: two large local outbreaks, namely legionella (Amsterdam, July 2006) and Q fever (West-Brabant province, outbreak period 2007–2009), and six national outbreaks, namely rubella (October 2004–January 2006), measles (March 2013–March 2014), meningococcosis W (March 2016–November 2017), hepatitis A (October 2016–November 2017) and mumps (two outbreaks periods: December 2009–January 2014 and April 2015–January 2016). In addition, specific guidance messages ((laboratory)inf@ct alerts) on laboratory diagnostic tests for psittacosis and on notification criteria for invasive group A streptococcal disease were identified in our study period and included.

Medians and means of these delays during the outbreak periods were calculated and compared with the delays for identical time periods before and after the outbreak period, using the permutation test. We observed a large increase in patient identification delay of 42 days during the first year of the Q fever outbreak. We excluded this delay from our analysis because this was an exceptional situation where the disease and diagnostic confirmation methodology was unknown to physicians and medical microbiologists at the time and patients were retrospectively diagnosed with extreme delay.

Alerts regarding pandemic influenza A(H1N1) were not included as the disease was only temporarily notifiable and no comparison with delays before and after the outbreak could be made.

### Software

Delays were analysed using SPSS (version 24). For statistical analyses of delays across periods, R (version 3.5.1, R Foundation for statistical computing, Vienna, Austria) was used.

### Ethical statement

In accordance with Dutch law, no informed consent was required for this study using anonymised routine surveillance data.

## Results

In total, 144,066 notifications of 28 different infectious diseases were included: 50,541 in period 1, 47,163 in period 2 and 46,362 in period 3. Numbers of included notifications per disease per period, numbers of cases for which delays could be calculated, median disease identification delay D1X, notification delay D3 and reporting delay D6, per disease and for all cases are displayed for each period in Table 1. For all three delays, the medians for all cases together decreased over time (D1X from 29 to 23 days, D3 from 2 to 0 days, D6 from 1 to 0 days). The mathematical averages of median delays of all individual diseases decreased as well, but to a lesser extent (Table 1).

**Table 1:** Median disease identification delay, notification delay and reporting delay in days, per infectious disease per period, and comparison of delays in periods 1–3, the Netherlands, July 2003–November 2017 (n = 144,066)

Infectious disease	Period 1 (2003–2008)				Period 2 (2009–2012)							Period 3 (2013–2017)						
	Number	D1X	D3	D6	Number	D1X	Diff^a^	D3	Diff^a^	D6	Diff^a^	Number	D1X	Diff^a^	D3	Diff^a^	D6	Diff^a^
Mumps	NR	NA	NA	NA	1,657	9	NA	0	NA	0	NA	445	9	NA	0	NA	0	NA
Botulism	10	9.5	0^b^	0	2	25.5	16	0	0	0	0	2	17	7.5	1	1	0,5	0.5
Brucellosis	35	31	2.5^c^	1	12	38	7	1	−1.5	0.5	−0.5	23	55	24	0	−2.5	0	−1
Cholera	17	13	0^b^	0	10	8.5	−4.5	0	0	0.5	0.5	5	9	-4	0	0	0	0
Diphtheria	0	NA	NA	NA	2	23.5	NA	0	NA	0	NA	8	16	NA	0	NA	0	NA
Group A streptococcal disease	NR	NA	NA	NA	839	4	NA	0	NA	0	NA	972	4	NA	0	NA	0	NA
Hantavirus disease	NR	NA	NA	NA	57	22.5	NA	0	NA	0	NA	130	19	NA	1	NA	0	NA
Hepatitis A	1,500	8	0^b^	0	683	8	0	0	0	0	0	703	6	−2^a^	0	0	0	0
Hepatitis B	1,401	11	2^b^	1	655	12	1	1	−1	0	−1^a^	520	11	0	0	−2^a^	0	−1^a^
Invasive *Haemophilus influenzae* type b disease	NR	NA	NA	NA	86	6	NA	0	NA	0	NA	109	6,5	NA	0	NA	0	NA
Invasive pneumococcal disease	NR	NA	NA	NA	191	5	NA	1	NA	0	NA	188	5	NA	0	NA	0	NA
Legionellosis	1,759	6	0^c^	0	1,347	6	0	0	0	0	0	2,091	6	0	0	0	0	0
Leptospirosis	172	23	6^c^	1	122	19	−4	3	−3^a^	0	−1^a^	385	14	−9^a^	1	−5^a^	0	−1^a^
Listeriosis	NR	NA	NA	NA	279	6	NA	0	NA	0	NA	430	5	NA	0	NA	0	NA
Malaria	1,487	4	4^c^	1	937	5	1	1	−3^a^	0	−1^a^	1,222	5	1	0	−4^a^	0	−1^a^
Meningococcal disease	1,212	2	0^b^	0	505	3	1	0	0	0	0	611	4	2^a^	0	0	0	0
Measles	134	6	0^b^	0	95	9	3^a^	0	0	0	0	2,838	6	0	0	0	0	0
CA-MRSA infection	NR	NA	NA	NA	42	33.5	NA	4	NA	1	NA	37	13	NA	6	NA	1	NA
Psittacosis	305	31	2^c^	1	269	29	−2	1	−1^a^	0	−1^a^	247	11	−20^a^	0	−2^a^	0	−1^a^
Paratyphoid A/B/C	187	13	1^b^	0	156	18	5	0	−1	0	0	172	22	9^a^	0	−1	0	0
Pertussis	37,524	36	2^b^	1	31,455	35	−1	1	−1^a^	0	−1^a^	29,362	33	−3^a^	0	−2^a^	0	−1^a^
Q fever	1,208	31	2^c^	1	3,005	22	−9^a^	2	0	0	−1^a^	106	25	−6	0	−2^a^	0	−1
Rubella	421	13	1^c^	0	11	10	−3	0	−1	0	0	61	6,5	−6.5^a^	0	−1	1	0
Shigellosis	2,053	11	2^b^	0	2,279	13	2^a^	1	−1	0	0	2,123	14	3^a^	0	−2	0	0
Tetanus	NR	NA	NA	NA	10	2	NA	1	NA	0	NA	4	11	NA		NA	0	NA
Typhoid fever	160	12	1^b^	0	87	13	1	0	−1	0	0	97	12	0	0	−1	0	0
Food-borne infections	521	8	1^b^	1	174	NA	NA	NA	NA	1	0	150	NA	NA	NA	NA	1	0
STEC infection	435	8	1^b^	0	2,196	12	4^a^	1	0	0	0	3,321	12	4^a^	0,5	−0.5	0	0
**Total: median delay of all cases per period (n cases)**	**(50,541)**	**29 (44,535)**	**2 (50,395)**	**1 (50,437)**	**(47,163)**	**27** **(35,294)**	NC	**1 (44,448)**	NC	**0 (47,011)**	NC	**(46,362)**	**23** **(32,193)**	NC	**0** **(40,262)**	NC	**0** **(46,312)**	NC
**Average of median delays of infectious diseases (n diseases)**	**(19)**	**14.6**	**1.4^d,e^**	**0.5**	**(28)**	**14.7**	NC	**0.7**	NC	**0.1**	NC	**(28)**	**13.2**	NC	**0.4**	NC	**0.1**	NC

CA-MRSA: community-acquired meticillin-resistant *Staphylococcus aureus*; D1X: disease identification delay; D3: notification delay; D6: reporting delay; Diff: difference with period 1 (in days); NA: not applicable; NC: not calculated; NR: not reportable; STEC: Shiga toxin-producing *Escherichia coli*.

^a^ p value < 0.05.

^b^ Notifiable for physicians (group B).

^c^ Notifiable for laboratory (group C).

^d^ Average for group B diseases: 0.9 days.

^e^ Average for group C diseases: 2.1 days.

**Figure 2:**
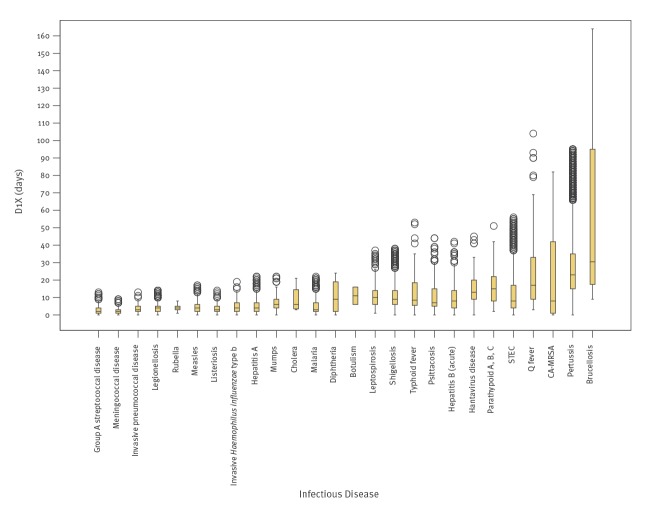
Medians and boxplot^a^ for disease identification delay per infectious disease for period 3, the Netherlands, January 2013–November 2017 (n = 46,362)

CA-MRSA: community-acquired meticillin-resistant *Staphylococcus aureus*; D1X: disease identification delay; IQR: interquartile range; STEC: Shiga toxin-producing *Escherichia coli*.

^a^ IQR: 25th–75th percentile in boxes, values between 1.5 IQR (lines) and outliers ( ͦ).

The disease identification delay was the longest delay and showed most variation between diseases, with medians ranging between 2 days (meningococcosis in period 1 and tetanus in period 2) and 55 days (brucellosis in period 3). The distribution of this delay per disease for period 3 is shown in Figure 2.

In order to visualise medians and IQR in the graph, extreme values > 3 IQR above the box were removed and, for brucellosis, only IQR is visualised. Tetanus is missing, as statistical analysis could not be performed on data of one case.

The median notification delay decreased in period 2 for most diseases (10/18, with 4/10 statistically significant), see Table 1. A significant decrease was observed for pertussis, malaria, leptospirosis and psittacosis. The shortening of the delay in ‘group C’ exceeded that in ‘group B’ diseases (averages 2.1 vs 0.9). In period 3, this delay decreased further (12/18 diseases, with 6/12 statistically significant). In 2016–2017, the percentage of cases notified more than 3 days after laboratory confirmation had substantially decreased compared with period 1, as calculated by Reijn [1]. This percentage decreased for shigellosis from 42.0% to 11.9%, for Shiga toxin-producing *Escherichia coli* (STEC) from 33.3% to 16.9%, for measles from 15.7% to 12.5%, for typhoid fever from 22.3% to 14.3% and for hepatitis A from 20.9% to 4.6%.

The median reporting delays also showed a clear decrease in period 2 (medians of 7/19 diseases decreased, 6/7 statistically significantly). Malaria, psittacosis and pertussis, for which the legal timeframe for reporting to the RIVM was adjusted, were reported significantly faster (p < 0,05), see Table 1. For the other diseases, the reporting delay did not decrease. In period 3, no further decrease in median delays was observed.

**Figure 3:**
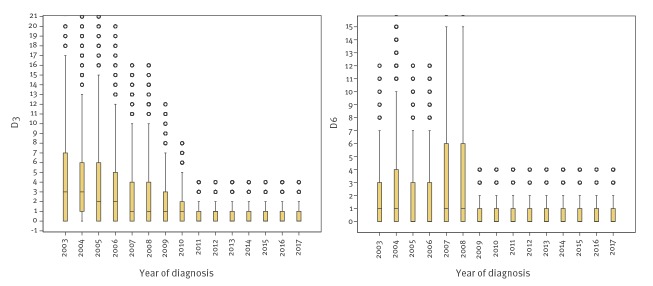
Median and boxplot^a^ notification delay and reporting delay, per year of diagnosis per disease, the Netherlands, July 2003–November 2017 (n = 144,066)

D3: notification delay; D6: reporting delay.

^a^ IQR: 25th–75th percentile in boxes, values between 1.5 IQR (lines) and outliers ( ͦ).

When displayed per year, a gradual shortening was observed for the notification delay from the beginning of the study period (July 2003) until 2012. For the reporting delay, the main decrease was in 2009, the year following the new law (Figure 3).

Extremes and outliers > 20 were removed to allow the medians and interquartile ranges to be visible in the graph.

### Timeliness according to the legal timeframe

In 2016–2017, the performance threshold of at least 80% timely notification was met, as 82.3% of all 14,447 included notifications were made within 1 working day (Table 2). This was an important improvement compared with 2008, the last year of the former law, when only 51.3% of notifications were received in time. In 2016–2017, the threshold was reached for 20 of 28 diseases. The notification timeliness for eight diseases did not fulfil the threshold at that time. The overall reporting delay was also timely, and even better than the notification delay, as 98.4% (14,044/14,810) of total cases were reported to the RIVM in time, and almost all diseases (26/28) were reported timely according to the legal timeframe of 1, 3 or 7 days.

**Table 2:** Timeliness of notified cases according to legal threshold, the Netherlands, 2016–2017 (n = 29,491)

Infectious disease	Timeliness ≤ 1 day			Timeliness ≤ 3 days		
	Number	In time	D3 (%)	Number	In time	D6 (%)
Mumps	88	80	90,9	97	94	96.9^a^
Botulism	2	1	50	2	2	100^b^
Brucellosis	8	7	87,5	8	7	87.5^a^
Cholera	1	1	100	1	1	100^a^
Diphtheria	3	2	66.7	3	2	66.7^b^
Group A streptococcal disease	367	317	86.4	418	409	97.8^a^
Hantavirus disease	68	51	75	78	77	98.7
Hepatitis A	366	339	92.6	392	383	97.7
Hepatitis B (acute)	157	126	80.3	172	165	95.9^a^
Invasive *Haemophilus influenzae* type b disease	45	37	82.2	49	45	91.8
Invasive pneumococcal disease	66	53	80.3	73	70	95.9
Legionellosis	868	809	93.2	940	925	98.4
Leptospirosis	145	93	64.1	160	157	98.1^a^
Listeriosis	171	155	90.6	185	181	97.8
Malaria	392	287	73.2	404	390	96.5^a^
Meningococcal disease	240	223	92.9	307	303	98.7
Measles	16	13	81.3	18	15	83.3^b^
CA-MRSA infection	11	4	36.4	11	6	54.4
Psittacosis	83	69	83.1	98	92	93.9
Paratyphoid A	19	16	84.2	21	20	95.2
Paratyphoid B	50	40	80	55	54	98.2
Paratyphoid C	3	3	100	3	3	100
Pertussis	9,598	7,843	81.7	9,794	9,717	99.2^a^
Q fever	33	26	78.8	35	31	88.6
Rubella	1	1	100	1	1	100^b^
Shigellosis	746	629	84.3	774	752	97.2
Tetanus	0	NA	NA	0	NA	NA
Typhoid fever	28	23	82.1	33	33	100
Food-borne infections	0	NA	NA	0	NA	NA
STEC infection	872	638	73.2	912	875	95.9
**Total**	**14,447**	**11,886**	**82.3**	**15,044**	**14,810**	**98.4**
**Average across all infectious diseases (%), n = 30**	**NA**	**NA**	**81.1**	**NA**	NA	**93.7**

CA-MRSA: community-acquired meticillin-resistant *Staphylococcus aureus*; D3: notification delay, within 1 working day; D6: reporting delay, within 1, 3 or 7 days; NA: not applicable; STEC: Shiga toxin-producing *E. coli*.

^a^ Within 7 days.

^b^ Within 1 day.

### Timeliness according to other timeframes

Timeliness of infectious diseases notification with regard to serial interval was good: medians of total local delay for six of eight diseases were within the serial interval in period 3, and the threshold of 80% notifications within the serial interval was reached for five of seven diseases in 2016–2017 (Table 3). Also, medians were below one or two incubation times (6/10 and 7/10, respectively) for the majority of diseases in period 3, while the threshold was reached to a lesser extent in 2016–2017 (4/9 and 5/9, respectively). Regarding outbreak control timeframes, only medians of hepatitis A and B and measles fulfilled the outbreak control condition in period 3. The percentages of timely notified measles was 72.2 and therefore close to the 80% thresholds of sufficient timeliness. Only 49% of mumps cases were within the outbreak control timeframe and therefore insufficient.

**Table 3:** Total local delay median in period 3 (2013–2017; n = 11,311) and cumulative percentage in period 2016–2017 (n = 9,066), per infectious disease, the Netherlands

Infectious disease	Number of cases		Serial interval			Within 1× incubation period			Within 2× incubation periods			Outbreak control timeframe		
	P3	2016–17	Median in days (SD)	P3 median^a^	2016–17 cum%^b^	Incubation period (1)	P3 median^a^	2016–17 cum%^b^	Incubation periods (2)	P3 median^a^	2016–17 cum%^b^	P	P3 median^a^	2016–17 cum%^b^
EHEC/STEC infection	2,140	684	NA	NA	NA	3,5	12	7.7	7	12	27.3	NA	NA	NA
Hepatitis A	664	229	27.5 (4)	**7**	**94.4**	14	**7**	**84.5**	28	**7**	**94.4**	**17**	**7**	**90.1**
Hepatitis B	490	162	47.5 (20)	**12**	**88.9**	75	**12**	**94.4**	150	**12**	**97.5**	**42**	**12**	**88.3**
Measles	2,724	18	11.6 (2,4)	**4**	**88.9**	10	**4**	**88.9**	20	**4**	**88.9**	**5**	**4**	72.2
Meningococcal disease	603	303	14	**3**	**95**	3.5	**3**	71.6	7	**3**	**87.1**	NA	NA	NA
Mumps	436	96	19.1 (5,4)	**9**	**91.7**	17	**9**	**87.5**	34	**9**	**100**	**8**	9	49
Pertussis	2,118	6,819	16 (13)	34	15.1	8,5	34	5.3	17	34	16.8	**4.5**	34	1.7
Rubella	61	1^c^	18.3 (range: 15–23)^d^	**7**	NC^c^	15	**7**	NC^c^	30	**7**	NC^c^	NA	NA	NA
Shigellosis	1,979	722	5 (3.5)	14	4.2	2	14	0.4	4	14	2.6	**3**	14	1.4
Typhoid fever	96	33	NA	NA	NA	11	12	36.4	22	**12**	69.7	NA	NA	NA
Typhoid fever	96	33	NA	NA	NA	11	12	36.4	22	**12**	69.7	NA	NA	NA

Cum: cumulative; D1: local delay; EHEC: enterohaemorrhagic *Escherichia coli;* STEC: Shiga toxin-producing *E. coli*; NA: not available. NC: not calculated; P: reporting delay median needed for PIR2 = 1/R; P3: period 3, 2013–2017; SD: standard deviation.

Numbers in bold: notification within timeframe (serial interval, incubation period(s) or outbreak control timeframe) or above threshold (≥ 80%).

^a^ Median (days) across infectious disease as retrieved from Table 1.

^b^ Cumulative percentage per disease notified within the serial interval, incubation period(s) or outbreak control timeframe (D1).

^c^ Not included, as number is too small.

^d^ According to Vink et al. [10]

### Influence of alert systems on timeliness: signalling reports and (lab)inf@ct

**Figure 4:**
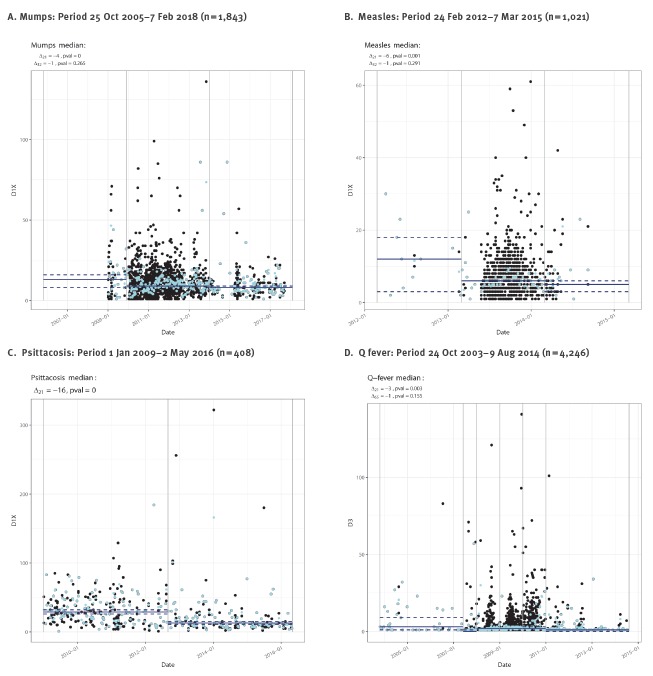
Change of median disease identification delays or notification delays in response to outbreak alerts (mumps, measles, Q fever) or specific guidance (psittacosis), the Netherlands

D1X: disease identification delays; D3: notification delay.

Increased awareness through signalling reports and (lab)inf@cts contributed to a significant decrease in the median disease identification delay during the mumps outbreak starting in December 2009 (4 days), and for measles (6 days) and psittacosis (18 days). The median notification delay for Q fever decreased by 3 days after information was provided to professionals in June 2007, p < 0.05 (Figure 4). For the other outbreaks, disease identification time and notification delay did not change significantly after alerts were given.

Δ21: period before the outbreak (mumps, measles, Q fever) or period after alert for guidance (psittacosis); Δ32: period before the outbreak.

Black dots: observed delays; blue dots: median delay per week; horizontal blue line: median of period; dashed horizontal blue line: confidence interval of median of period.

## Discussion

This study analysed delays in the notification and reporting chain of infectious diseases in the Netherlands in the period 2003–2017. We observed that legal adjustments for mandatory notification to the MHS and reporting to the RIVM led to shorter delays.

We show that the law adjustment successfully reduced the reporting delay to the RIVM and that MHS are capable of swiftly adjusting their reporting methodology. In our opinion, the decrease of reporting delay observed in 2009 was mainly the result of legal adjustments, as the electronic reporting system between MHS to RIVM, in place since 2003, did not alter. However, the legal adjustments led to renewed attention towards monitoring delays, which in our opinion probably contributed as well. Nowadays, MHS overall fulfil the thresholds for legal timeframes, with at least 80% of cases reported in time in 2016–2017.

### Notification delays

Notifications by physicians and laboratories to the MHS are now also timely. We observed a steady reduction in the median delay by 1 day every 2 years, already starting in period 1. While notification systems involving laboratories generally lead to more timely notifications than those involving only physicians [5], we observed a longer average notification delay for diseases notifiable for laboratories than for diseases notifiable for physicians in period 1. The gradual decrease since 2003 was probably related to local agreements between MHS, physicians and microbiologists on anonymous pre-notifications for group B diseases by the laboratory. This was recommended under the former law to reduce reporting delay in group B diseases [13]. In our opinion, the variety of notification procedures explains why a substantial delay reduction was only achieved in 2011. In 2016–2017, for all six diseases studied by Reijn et al., the percentage of cases notified more than 3 days after laboratory confirmation was substantially reduced, and legal thresholds were achieved for at least 80% of cases for D3 (82.3%). Nevertheless, for some diseases, notifications by the involved physicians and laboratories still need to become more timely, namely botulism, diphtheria, hantavirus infections, leptospirosis, malaria, and infections with community-acquired meticillin-resistant S*taphylococcus aureus* and STEC. These diseases have in common that they are rare and may need additional laboratory tests after initial confirmation, which are performed by specialised reference laboratories leading to delay in notification. For STEC infections, this delay is of special concern as early identification of a common source is important. We recommend that MHS monitor notification delays in their region and identify ways of improvement together with local laboratories and involved physicians.

Although we attribute the shortening of notification delays over time mainly to the legal adjustment in December 2008, other developments such as faster notification systems probably contributed as well. Laboratories nowadays notify mainly through automated electronic systems which in comparative studies have proven faster than conventional methods [5]. Also, MHS state that most notifications are performed by laboratories nowadays. Another influence may have been the quarterly feedback of notification delays that RIVM has provided to the MHS since 2006, as it can be used by MHS to monitor and evaluate notification timeliness and in their communication with health professionals. Nevertheless, these developments have only contributed to shorter notification delays since the legal adjustment obliging laboratories to notify in addition to physicians. Lastly, we did not see major changes of D3 and D6 between period 2 and 3.

Other countries also observed shorter reporting delays after law adjustments. In Germany, the median local reporting time to state health departments decreased from 4 to 1 day after adjusting the legal threshold from 1 week to 1 day [3]. In the United Kingdom (UK), after introduction of a new legal obligation for laboratories to report a specified list of causative agents, the median notification delay by laboratories (D2) decreased from 10 to 8 days, fulfilling the timeframe of 21 days. However only a minority of laboratories reported more than 90% of cases timely [4]. An international systematic literature review of publications on timeliness of notification systems, published between 2000 and 2017, revealed that notification delay at local level was evaluated most frequently [5]. Timeframes for notification varied between the included studies, but the most common predefined timeframe, either legal or defined for the study itself, was within 48 hours. Timeliness of notification systems was sufficient in only a minority of studies. Notifications by laboratories and by laboratories combined with notifications by physicians, as in the Netherlands, were related to more timely notifications [5,14]. Short delays, as those achieved in the Netherlands, are also observed in other European countries: both Germany and the UK have reported a majority of notifications arriving at LHD within 1 day [5,15-18].

Increased awareness during outbreaks and provision of guidance on laboratory testing and notification criteria shortened disease identification and notification delays for some diseases. Although not applicable for all diseases, we demonstrate that disease identification can be expedited, which is especially important when reporting and notification delays have been minimised and the disease identification delay dominates in the notification chain. This is particularly relevant for measles and mumps, but also in case of a newly emerging infectious disease.

We show that interventions such as law adjustments and raising awareness can decrease notification and reporting delays, but thresholds for outbreak control are not yet achieved.

### Disease identification delays

The average across the median identification delays of the individual disease decreased in the third period by 1.5 day compared with period 1 and 2. Five diseases showed a significant decrease in period 3 compared with period 1, while four diseases showed a significant increase. We could not identify a clear trend and therefore not generate hypotheses on causes for the overall decrease in period 3. Insight in patient, doctor and laboratory delay would facilitate developing hypotheses on factors that could have contributed to changes in this delay.

### Other timeframes

Disease-specific timeframes are still a concern. In the Netherlands, thresholds for notification within two incubation periods still are not met for bacterial pathogens causing gastrointestinal diseases such as enterohaemorrhagic *Escherichia coli* and STEC, *Shigella* and *Salmonella typhi* fever, which has been observed before [1]. In our opinion, this is related to a short incubation period in combination with patient delay in case of mild disease and doctor delay for not directly initiating laboratory testing. Timeframes for outbreak control involving total local delay (D1) were only met for hepatitis A, hepatitis B and measles, the latter meeting the timeframe for the first time in the period 2013–2017. This was probably a consequence of the outbreak in 2013–2014, when the disease identification delay was reduced by, among other things, the RIVM alert systems. The performance threshold of measles was close to the threshold of 80%. Therefore, we advise to further decrease patient, doctor and laboratory testing delays, especially for bacterial gastroenteritis and measles. Given the current measles outbreaks in Europe and regular imported cases in the Netherlands, we recommend enhancing doctors’ awareness and optimising laboratory confirmation procedures to achieve early detection of measles cases for optimal outbreak control [19]. As we have demonstrated in this study, the RIVM alert systems can contribute to achieve this.

### Strengths

This is the first study in the Netherlands analysing timeliness of notifications, describing the effect of the law change and of alerts and guidance provided during an outbreak, and including notifications of almost 15 years. To our knowledge, studies systematically analysing the effect of alerts and guidance have not been performed before.

### Limitations

Our study did not investigate the way these changes in delays were achieved on local level. Although notification is mandatory for both physicians and heads of laboratories, it is, according to the MHS, mainly laboratories that perform notifications. This study did not provide best practices of laboratories to achieve legal thresholds.

Although delay of disease identification is the longest delay in the notification and reporting chain, we cannot determine patient, doctor or laboratory delay because information on the first date of consultation (T_C_) or of requesting laboratory testing (T_L_) is not available, as this is not legally required. We expect that the ratios between these delays differ by disease, as some diseases develop gradually (resulting in patient delay), are nonspecific (resulting in doctor delay) or may need laboratory tests which are not available at every laboratory, or two-point serology testing (resulting in laboratory delay). During outbreaks, public health professionals need real-time information on new cases to monitor the effect of control measures. Insight in patients, doctors and laboratory delays is necessary to decide whether and how these delays in the notification chain can be reduced. The importance of this information has been emphasised before for pertussis surveillance and control in the Netherlands [20].

In some countries such as Sweden, the UK and the United States, specimen collection dates are recorded in the laboratory surveillance system, which gives an indication of laboratory delay [4,5,21]. However, dates of doctor’s consultation and laboratory test initiation are not routinely collected in European Union countries (personal communication: M Diercke, Robert Koch Institute, February 2019; AM O’Connor, Public Health England, February 2019; A Jacks, Public Health Agency Sweden, February 2019). We recommend including these time points in every notification to enable monitoring of causes of delay before laboratory confirmation and measuring the effect of raising awareness among public and physicians during outbreaks. Although additional data in surveillance systems should, in order to maintain compliance by reporting health professionals, not be requested lightly, insight in these time points is important. Therefore, it is worthwhile to investigate how these data can be collected automatically in the electronic reporting systems at a minimal workload for the notifying health providers. As an alternative, these data can be collected only when specifically needed during outbreaks when healthcare providers will be more motivated to provide this information.

Another limitation is missing data on the completeness of notification in the Netherlands. Incompleteness of notified infectious diseases is an even larger concern for public health than delayed notification. Completeness rates for laboratory-confirmed hospitalised pertussis cases have been determined as low as 16.5–22% for cases 2 years and older and between 52–61% for children younger than 2 years [22]. Reporting completeness during the measles outbreak in 2013-2014 has been estimated as low as 9%, although this is mainly the result of patients not seeking medical care (underascertainment) [23]. Better insight in notification completeness is necessary, at local as well as national level, in order to improve the surveillance system.

### Conclusions

Adjustments in the law regulating infectious disease control successfully reduced notification delays by physicians and laboratories to the MHS and reporting delays to the RIVM. Legal timeliness thresholds overall were achieved, although notification delays can still be shortened for some diseases and therefore need to be monitored by the MHS. To achieve outbreak control thresholds, also disease identification delays need to be reduced, which especially applies for measles and bacterial gastroenteritis. We recommend including dates of doctor’s consultation and laboratory request into notification records to determine patient, doctor and laboratory delays during outbreaks of emerging infectious diseases.
